# Applications of Coarse-Grained Models in Metabolic Engineering

**DOI:** 10.3389/fmolb.2022.806213

**Published:** 2022-03-08

**Authors:** Dieu Thi Doan, Manh Dat Hoang, Anna-Lena Heins, Andreas Kremling

**Affiliations:** ^1^ Systems Biotechnology, TUM School of Engineering and Design, Technische Universität München, Garching, Germany; ^2^ Biochemical Engineering, TUM School of Engineering and Design, Technische Universität München, Garching, Germany

**Keywords:** mathematical modeling, resource allocation, kinetic model, metabolic engineering, systems biotechnology, metabolic and gene regulatory networks, microbial growth

## Abstract

Mathematical modeling is a promising tool for better understanding of cellular processes. In recent years, the development of coarse-grained models has gained attraction since these simple models are able to capture and describe a broad range of growth conditions. Coarse-grained models often comprise only two cellular components, a low molecular component as representative for central metabolism and energy generation and a macromolecular component, representing the entire proteome. A framework is presented that presents a strict mass conservative model for bacterial growth during a biotechnological production process. After providing interesting properties for the steady-state solution, applications are presented 1) for a production process of an amino acid and 2) production of a metabolite from central metabolism.

## 1 Introduction

To gain a full understanding of cellular processes, the usage of mathematical modeling and the analysis of those have become a standard in metabolic engineering, systems biology, and process engineering. Predictive models which can describe relevant cellular processes can be used as a basis for process observation and process design with the intention to optimize the properties and behavior of the cells. Even though bacteria are very diverse, the basic principles of their metabolism are quite similar. In general, every bacterial population has to cope with its environment, scavenge for nutrients, and then coordinate its central metabolism accordingly for growth and survival. The underlying regulatory networks are very densely intertwined, large, complex, and not fully known or incompletely understood, thus providing a big challenge to understand the processes in its entirety. Mathematical models trying to describe all these processes in detail are challenging and nearly impossible because of the huge number of variables and uncertain parameters.

Coarse-grained models have been used in the recent years and are now frequently used to get a better understanding on cellular control strategies, gene expression, and resource allocation Bollenbach et al. (2009), Scott et al. (2010). In this type of model, levels of cellular organization with similar functions are “lumped” together into a small number of modules Maitra and Dill (2015), Giordano et al. (2016), Pandey and Jain (2016), Sharma et al. (2018), Molenaar et al. (2009). In contrast to whole-cell models with hundreds of individual reactions and components, the number of state variables in coarse-grained models is very low, and kinetic parameters are obtained by either a rough estimation from literature data or by regression from experimental data. An important hallmark of coarse-grained models is allocation of cellular resources. This is expressed, for example, by linking biochemical reactions to the available fraction of the proteome for the respective module. In this way, a reaction can only take place if enough resources are available. The goal can be achieved by efficient proteome allocation in a way where no resources are wasted. Using this fundamental assumption, many coarse-grained models have been proposed to analyze certain metabolic effects such as metabolic overflow Basan et al. (2015), production of heterologous protein Scott et al. (2010), or applications in synthetic biology Weiße et al. (2015).

Typically, coarse-grained models are written down as a set of differential equations for the components of the model, whose unit, for example, is the number of molecules per cell or mol/g dry cell weight. However, mass balance equations must fulfill the conservation of mass as dictated by the first fundamental theorem of thermodynamics, and often, a consistent transfer from mass balance equations to differential equations for the concentrations of the model components is faulty or inadequate. Therefore, we start by a brief recapitulation of the structure of the ordinary differential equations for coarse-grained models that are combined with models for the environment, for example, in a bioreactor process system. Here, a new relationship for the specific growth rate in dependence on the exchange reactions of the entire network is given. This equation is fundamental since it guarantees strict mass conservation for the complete system. Conventionally, the growth rate is an empirical function and, therefore, strict mass conservation is not ensured. In a second step, we analyze the model and show interesting properties of the steady-state behavior. We provide a general steady-state solution for biochemical networks and compare outcomes of a traditional flux balance analysis with our new approach. Finally, various applications and extensions for a broad spectrum of problem formulations in biotechnology are provided: 1) an L-phenylalanine production process and 2) the production of a metabolite from central metabolism. Hereby, problems of resource allocation as well as problems of parameter estimation are addressed.

## 2 Mass Conversation in Models for Microbial Systems

From thermodynamic principles, mass conversion is crucial and plays the major role of determining the time course of selected quantities of interest (system volume, concentration of reaction partners, and temperature) which are called state variables. From a static view on the biochemical reaction equations alone, however, it is not possible to infer on the time course of the state variables. A mass balance equation that describes the change of a compound over time and sums up the material flow in and out of the system comes into play here. It is a differential equation. Since we are interested in the mass *m*_*i*_ of a component *i*, the mass balance reads as

$$\frac{d\;m_{i}}{dt}\;\;=\;\;J+P.$$ (1)

In this general equation, *J* describes the mass flow into the system while *P* describes conversion inside the system, for example, by biochemical reactions. Both summands depend on other state variables in the system. In the current form, the equation cannot be applied. The reason is as follows: for biochemical reaction networks, *P* describes mass conversion by reactions, and the reaction velocity strongly depends on the concentration of a compound given by *c*_*i*_ = *m*_*i*_/*V* of the reaction partners and not on the mass *m*_*i*_ of the reaction partners alone. For applications in systems biology, synthetic biology, and biotechnology, a different convention is used for the definition of the concentration of the cellular components (but not for environmental compounds). Since it is much easier to determine the entire biomass *m*_*X*_ than the cellular volume, the following definition is used, instead, for the concentration of an intracellular metabolite: *c*_*i*_ = *m*_*i*_/*m*_*X*_.

To avoid inconsistencies, it is recommended to always start from the mass balance and reformulate the mass balance into an equation for the concentration (the resulting equation is not a mass balance in the strict sense, but in literature, we often find this term). For a cellular network, the basic differential equation then reads^1^

$$\dot{\underline{c}}\;\;=\;\;N\;\;\underline{r}\;-\;\mu \;\;\underline{c},$$ (2)

for the vector $\underline{c}$ , for the concentration of all components, a reaction system given by the stoichiometric matrix *N* and a rate vector $\underline{r}\left(\underline{c}\right)$ that is dependent on $\underline{c}$ as well. The specific growth rate *μ* is an integral parameter that—in a strict sense—is determined by the mass exchange of the population with its environment. Therefore, it is defined as

$$\mu \;={\dot{m}}_{X}/m_{X}.$$ (3)

The specific growth rate is related to the doubling time *τ* of the population in the relationship *τ* =  ln 2/*μ*. For the biomass itself, also, a mass balance is set up that takes into account the changes of biomass concentration due to removal from the bioreactor and biomass formation due to growth. However, a different approach can be used as shown in the *Supporting Information* that describes the changes of biomass based on the changes of all compounds representing the biomass. Therefore, from a formal point of view, the specific growth rate *μ* only depends on the rate vector $\underline{r}$ that describes internal processes as well as mass exchange with the surrounding and is given with the vector of all molecular weights of the components $\underline{w}$

$$\mu \;\;={\underline{w}}^{T}\;\;N\;\;\underline{r}.$$ (4)

Plugging Eq. 4 into Eq. 2, the latter can be rewritten as

$$\dot{\underline{c}}\;\;=\;\;N\;\;\underline{r}\;-\;{\underline{w}}^{T}\;\;N\;\;\underline{r}\;\;\underline{c}\;\;=\;\;\left(Id\;-\;\underline{c}\;\;{\underline{w}}^{T}\right)\;\;N\;\;\underline{r}\;\;=\;\;W\;\;N\;\;\underline{r},$$ (5)

with *W* representing the mass matrix. Together with the equations for the concentration for a substrate *S* and biomass *X* in a bioreactor system with feeding rate *q*_*in*_, feed concentration *S*_*in*_, and stoichiometric vector ${\underline{n}}_{S}$

$$\dot{S}\;\;=\;\;\frac{q_{in}}{V}\;\;\left(S^{in}\;-\;S\right)\;-\;{\underline{n}}_{S}^{T}\;\;\underline{r}X,$$ (6)

$$\dot{X}\;\;=\;\;\left(\mu \;-\;\frac{q_{in}}{V}\right)\;\;X,$$ (7)

the system is completely described. The first term of Eq. 6 accounts for the feeding substrate, the second the dilution due to the feed, and the last term the substrate uptake of the biomass. The second term of Eq. 7 represents the growth and a dilution due to the feed.

## 3 Steady-State Analysis

### 3.1 Flux Analysis

In classic flux balance analysis, the equation for a cellular network only consists of the stoichiometric matrix *N*. Solutions for rate vector $\underline{r}$ are investigated by determining the kernel of *N*, providing possible fluxes through the cellular network Orth et al. (2010). With the proposed approach, in addition, properties of the mass matrix *W* has to be taken into account as well, and the steady-state solution for the intracellular network is obtained from the relationship

$$0\;\;=\;\;W\;\;N\;\;\underline{r},$$ (8)

which is obtained from Eq. 5 by setting the left side to zero. The solution of this equation is determined by not only the kernel of *N* as in the classic flux balance analysis but also by the kernel of matrix *W*, which is determined by the molecular composition of the cell. The determinant of *W* is given by

$$\det\left(W\right)\;\;=\;\;1\;-\;\underset{i}{\sum }w_{i}\;\;c_{i}.$$ (9)

The addends of the second term are the mass fractions of cellular component *i*, and given the strict mass conservation, the sum over all mass fractions equals one. Therefore, the determinant of *W* is always zero, and *W* is nonsingular. Additionally, one can show that the kernel of *W* is in fact one-dimensional (the proof can be found in *Supplementary Information*). The complete solution ${\underline{r}}_{0}$ of the relationship given in Eq. 8 comprises two terms: the kernel of *N*, denoted by ${\underline{r}}_{n,0}$ , and second, the product of the Moore–Penrose inverse of *N*, denoted by *N* ^+^, and the kernel of *W*, denoted by ${\underline{c}}_{w,0}$

$${\underline{r}}_{0}\;\;=\;\;{\underline{r}}_{n,0}\;+\;N^{+}{\underline{c}}_{w,0}.$$ (10)

The two summands in the solution are not given in a unique way and can be written with scalar factor *s* and an arbitrary vector $\underline{a}$ with the same dimension as the rate vector ${\underline{r}}_{0}$ as follows:

$${\underline{c}}_{w,0}\;\;=\;\;\underline{c}\;\;s\;,\;s\in R\;\;$$ (11)

$${\underline{r}}_{n,0}=\left(Id-N^{+}N\right)\;\underline{a}.$$ (12)

**FIGURE 1:**
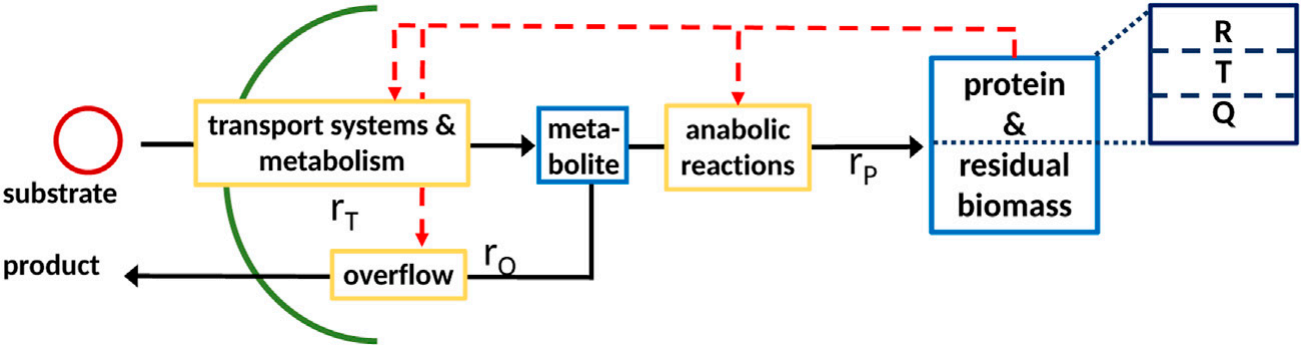
General scheme of a coarse-grained model with partitioned proteome (ribosomal proteins *R*, proteins linked with the central metabolism *T*, and residual protein fraction *Q*) as the self-replicator system Scott et al. (2014); it consists of two components, indicated as blue boxes; (metabolite, low molecular weight), protein; and residual biomass (high molecular weight; protein is assumed to be 50% of total biomass). The pools are connected by a minimal set of reactions, indicated by yellow boxes, for substrate uptake, overflow metabolism, and protein synthesis.

This principle holds true for all types of cellular networks independently of its size and form, which can range from whole-cell models to, in this case, coarse-grained models as shown in Figure 1.

A typical minimal reaction system as shown in Figure 1 is considered with a cellular network that represents the entire biomass (the sum of all components in the network weighted with their molecular weight) and only one anabolic reaction. The scheme is given as follows^2^:

$$\begin{array}{ll} \left(S\right)\;\; & \to \;\;\alpha \;\;M\\ \gamma \;\;M & \to \;\;\beta \;\;P\\ M & \to \;\;\left(\text{by}-\text{product}\right). \end{array}$$ (13)

It is to be noted that an extension to two or more anabolic reactions can be performed easily since, in general, the mass fractions of the macromolecules are well-known. The reaction systems, therefore, consist of a pool of metabolites *M*, proteins *P*, and reactions *r*_*i*_ that connect the pools with each other and the environment. Rate *r*_*T*_ describes the transport of the substrate into the cell, while rate *r*_*O*_ describes overflow metabolism. Proteins *P* are synthesized with rate *r*_*P*_ Maitra and Dill (2015), Giordano et al. (2016), Pandey and Jain (2016)) The vector of components reads $\underline{c}=\left(M,P\right)$ , and the stoichiometric matrix for this system is as follows:

$$N\;\;=\;\;\left(\begin{array}{ccc} \alpha & -\gamma & -1\\ 0 & \beta & 0 \end{array}\right).$$ (14)

Thus, the intracellular network for this minimal model can be written as

$$\dot{\left(\begin{array}{c} M\\ P \end{array}\right)}\;\;=\;\;N\;\;\left(\begin{array}{c} r_{T}\\ r_{P}\\ r_{O}. \end{array}\right)\;-\;\mu \;\;\left(\begin{array}{c} M\\ P \end{array}\right)\text{.}$$ (15)

For the basic structure, with Eq. 4, the specific growth rate is given by

$$\mu \;\;=\;\;\left(\alpha \;\;r_{T}\;-\;r_{O}\right)\;\;w_{M}+r_{P}\;\;\left(\beta \;\;w_{P}-\gamma \;w_{M}\right),$$ (16)

where $w_{M}$ and $w_{P}$ are the molecular weights of metabolites *M* and proteins *P*, respectively. With the observation from Eq. 10, the solution of the rate vector reads

$$\underline{r}\;\;=\;\;\left(\begin{array}{c} \frac{1}{\alpha^{2}+1}\\ 0\\ \frac{\alpha }{\alpha^{2}+1} \end{array}\right)a\;+\;N^{+}\underline{c}\;\;s$$ (17)

with the first term representing the solution from the stoichiometry of the system and the second term the solution determined by the molecular composition of the cell. With a closer look at solution Eq. 17, the rate connecting only intracellular components of the system, which is the protein synthesis rate *r*_*P*_, is only defined by the second term. Thus, for the assumption of the known specific growth rate *μ* and molecular composition of the cell, this rate is fixed, while the remaining rates, meaning the substrate uptake rate *r*_*T*_ and the overflow metabolism rate *r*_*O*_, are coupled through one degree of freedom *a*. If one of these rates is known, the degree of freedom *a* can be determined and, therefore, the last remaining rate.

### 3.2 Flux Analysis in Comparison to a Constraint-Based Method

**FIGURE 2:**
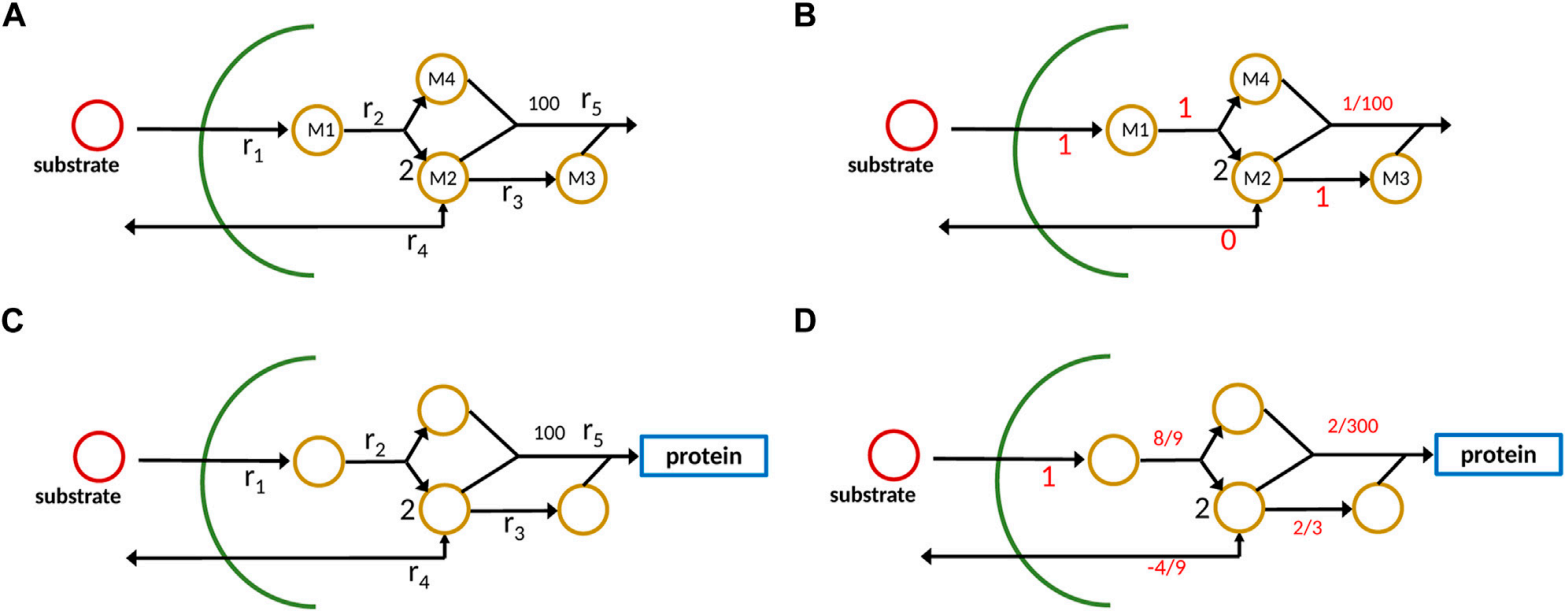
Example network with metabolites *M*_*i*_ and reactions *r*_*i*_; conventional representation **(A)**, new approach with one component representing protein/ biomass sector **(C)**. On the right side **(B,D)**, output data from the calculations are shown; it is to be noted that for the new approach, the dilution term must be taken into account which is not shown in the plot.

To illustrate the different outcomes when applying the new approach with strict mass conservation as seen in Eq. 5 in comparison to a standard analysis with a constraint-based method, a small network with four metabolites and five reactions is considered (Figure 2A).

Substrate *S* is taken up and four metabolites are generated which in the conventional approach are consumed in reaction *r*_5_ to produce biomass. To make it realistic, we assume that the stoichiometric coefficient for biomass is 100, that is, 100 small molecules are used to get 1 mol protein/ biomass. The reaction system is given as follows:

$$\begin{array}{ll} \left(S\right)\;\; & \to \;\;M_{1}\\ M_{1} & \to \;\;2\;\;M_{2}\;+\;M_{4}\\ M_{2} & \to \;\;M_{3}\\ M_{3} & \to \;\;\\ 100\;M_{2}\;+\;100\;M_{3}\;+\;100\;M_{4} & \to \left(\text{protein}/\text{biomass}\right). \end{array}$$ (18)

In the case of constraint-based models that are used, for example, for flux balance analysis or by Bollenbach et al. (2009), the stoichiometric matrix noted by *N*_1_ for the internal network has four rows and five columns:

$$N_{1}\;\;=\;\;\left(\begin{array}{ccccc} 1 & -1 & 0 & 0 & 0\\ 0 & 2 & -1 & 0 & -100\\ 0 & 0 & 1 & -1 & -100\\ 0 & 1 & 0 & 0 & -100 \end{array}\right).$$ (19)

Hence, the null space of *N*_1_ is one-dimensional, and the only possible solution, when providing 1 unit flux of substrate uptake, results in 0.01 unit of biomass (it is to be noted that in reaction 2, 2 mol of *M*_2_ are produced). The situation becomes different when strict mass conservation is taken into account. Here, we consider the formation of protein/biomass. Therefore, the overall biomass composition dictates the flux distribution in this case. From the depicted scheme, we infer the molecular weight for the components as follows (it is to be noted that these numbers are not unique, but used here for demonstration purposes) $\underline{w}\;=\;\left(w_{1},w_{1}/4,w_{1}/4,w_{1}/2,100\;w_{1}\right)$ . Given the stoichiometric matrix *N*_2_ (extension of *N*_1_ by one component for the protein, that is, one additional row) and the vector of molecular weights, the specific growth *μ* can be calculated as mentioned before with Eq. 4 with a possible flux vector $\underline{r}$ to be

$$\mu \;=\;\left(\begin{array}{ccccc} w_{1} & 0 & 0 & -w_{1}/4 & 0 \end{array}\right)\;\;\underline{r}.$$ (20)

It is to be noted that here only reactions that exchange with the environment (here *r*_1_ and *r*_4_) appear. In the case at hand, the stoichiometric matrix *N*_2_, taking into account the protein/ biomass fraction as additional component, has five rows and five columns and has full rank. However, the null space of *W* ⋅ *N*_2_ with *W* given in Eq. 5 is one-dimensional and represents the only possible flux distribution. Matrix *W* strongly depends on the cellular composition; the composition itself is the steady-state solution, if the system is given in the standard form (Eq. 2), and all reaction kinetics are known and well-parameterized. However, for the example, we choose a different way and start with a possible composition for biomass and back-calculate the fluxes for this case. Taking the following composition vector (mass fraction) $\underline{f}\;=\;\left(0.1,0.1,0.1,0.1,0.6\right)$ as an example, the resulting flux vector (scaled to 1 for the uptake rate) is $\underline{r}\;=\;\left(1,8/9,2/3,-4/9,2/300\right)$ . In this case, to fulfill all steady-state equations, an additional input flux is necessary (*r*_4_ is negative, that is, a second substrate is needed). This flux vector is very different from the solution not considering strict mass conservation.

### 3.3 Differential Algebra System

In the case of regulated systems, that are also named self-replicator systems, a superimposed control structure (shown in red in Figure 1) determines the allocation of protein resources in the individual reactions. The division of the entire proteome in fractions results in additional algebraic equations representing conservation conditions. This is shown exemplarily in Figure 1 with three fractions; fraction *R* represents ribosomes, fraction *T* represents transport and catabolism, and fraction *Q* represents the remaining proteins. The dependency of the rate for protein synthesis *r*_*p*_ on the ribosomal fraction *R* of the proteome is common to many approaches Scott et al. (2014). To derive a consistent system that can be used for numerical simulation, the resource allocation problem must be formulated in mathematical terms; here, we will describe two different approaches that result in a differential algebra system or in an optimization program.

First, the rate vector $\underline{r}$ of the minimal coarse-grained model is fixed with kinetic rate laws. For the rates involved in central metabolism, a dependency to the *T* fraction is applied, while rate *r*_*P*_ will depend on the *R* fraction. Furthermore, the drain from central metabolism will depend on metabolite *M*, while the transport reaction will depend on the main substrate *S*. The following rates are taken as examples for the case study:

$$\begin{array}{ll} r_{T}\;\; & =\;\;k_{T}\;\;\frac{S}{S+K_{T}}\;\;T\\ r_{P}\;\; & =\;\;k_{P}\;\;\frac{M}{M+K_{P}}\;\;R\\ r_{O}\;\; & =\;\;k_{O}\;\;M\;\;T. \end{array}$$ (21)

The dependencies of *T* and *R* from the entire proteome *P* are exploited from a data set that was published by Schmidt et al. (2016). The fraction of the *T* and *R* fractions is given in dependence on the specific growth rate *μ*. From the data, a linear relationship can be deduced. However, a direct implementation of functions *T*, *R* = *f*(*μ*) is not possible since *μ* itself depends on *R* and *T*. Therefore, we proceed as follows: for the case at hand, an algebraic system could be set up for the steady-state solution of the differential equation system. The system reads

$$\begin{array}{ll} \underline{0}\;\; & =\;\;N\;\;\left(\begin{array}{c} r_{T}\\ r_{P}\\ r_{O} \end{array}\right)\;-\;\mu \;\;\left(\begin{array}{c} M\\ P \end{array}\right)\\ 0\;\; & =\;\;\mu \;-\;{\underline{w}}^{T}\;\;N\;\;\underline{r}\\ 0\;\; & =\;\;T\;-\;f_{1}\left(\mu \right)\\ 0\;\; & =\;\;R\;-\;f_{2}\left(\mu \right). \end{array}$$ (22)

**FIGURE 3:**
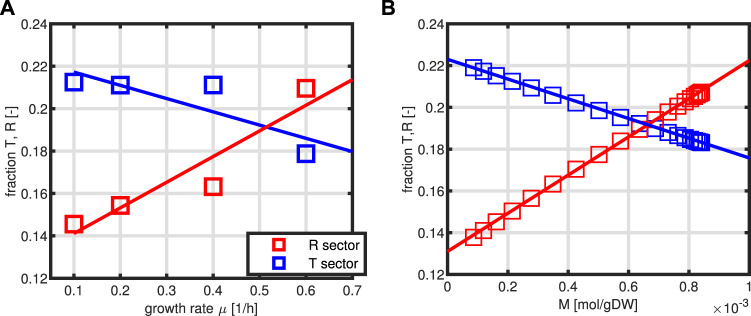
Relationship between the sectors *T* and *R* as a function of the specific growth rate *μ*. Experimental data are taken from the study by Schmidt et al., (2016) with protein representing 50% of the biomass **(A)**. Estimated kinetics of first order for *T*/*P* and *R*/*P* as a function of variable *M* **(B)**.

Thereby, the last two equations are determined by experimental data. From the solution for a broad range of the input variable, in our case, substrate concentration *S*, relationships of the form *T* = *g*_1_(*M*, *P*), and *R* = *g*_2_(*M*, *P*) are determined. In this way, also the dynamical system could be simulated. In addition, in this format, the system for the intracellular network consists only of two independent variables *M* and *P*. Figure 3 shows the dependencies of sectors *T* and *R* from the specific growth rate as measured experimentally (Figure 3A) and the kinetics for *T* and *R* as a function of metabolite *M* (Figure 3B).

### 3.4 Optimization Program

The first approach with fixed reaction kinetics is compared with an optimization program to check if the given experimental data for fractions *T* and *R* are optimal for the given specific growth rates. For this, we omit the determined dependencies given in the last subsection, and the following program is formulated:

$$\begin{array}{ll} \max_{R} & \Phi \text{,}\;\Phi \;\;=\;\;\mu \\ s.t. & \\ & \underline{0}\;\;=\;\;N\;\;\underline{r}\;-\;\mu \;\;\underline{c}\\ & \mu \;\;=\;\;{\underline{w}}^{T}\;\;N\;\;\underline{r}\\ & 0\;\;=\;\;P_{0}\;-\;T\;-\;R. \end{array}$$ (23)

**FIGURE 4:**
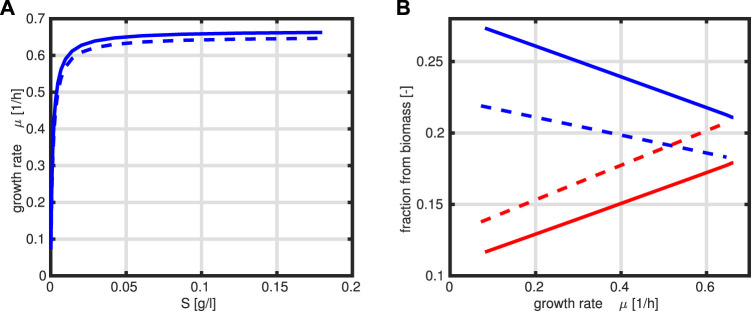
Specific growth rate *μ* as a function of substrate *S*, where the solid line represents the optimal case **(A)**. Fractions *R* (red) and *T* (blue) as a function of the growth rate *μ*, where the solid line represents the optimal case, and dashed lines are from presented data in Figure 3B.

The program is simplified to only one design variable *R* and the constraint that the sum of *R* and *T* is fixed to a constant value *P*_0_. The kinetic rate laws are taken as given above. Figure 4 compares the steady-state output of both approaches for a given range of the substrate concentration *S* (kinetic expressions and kinetic parameters are the same in both cases).

A comparison of the growth rate indicates that only a slightly higher growth rate could be achieved in the optimal case. This is based on the observation that the protein fraction that is allocated to the *T* fraction is always higher than that for the *R* fraction which is not the case in Figure 3B.

## 4 L-phenylalanine Production With *Escherichia coli*

**FIGURE 5:**
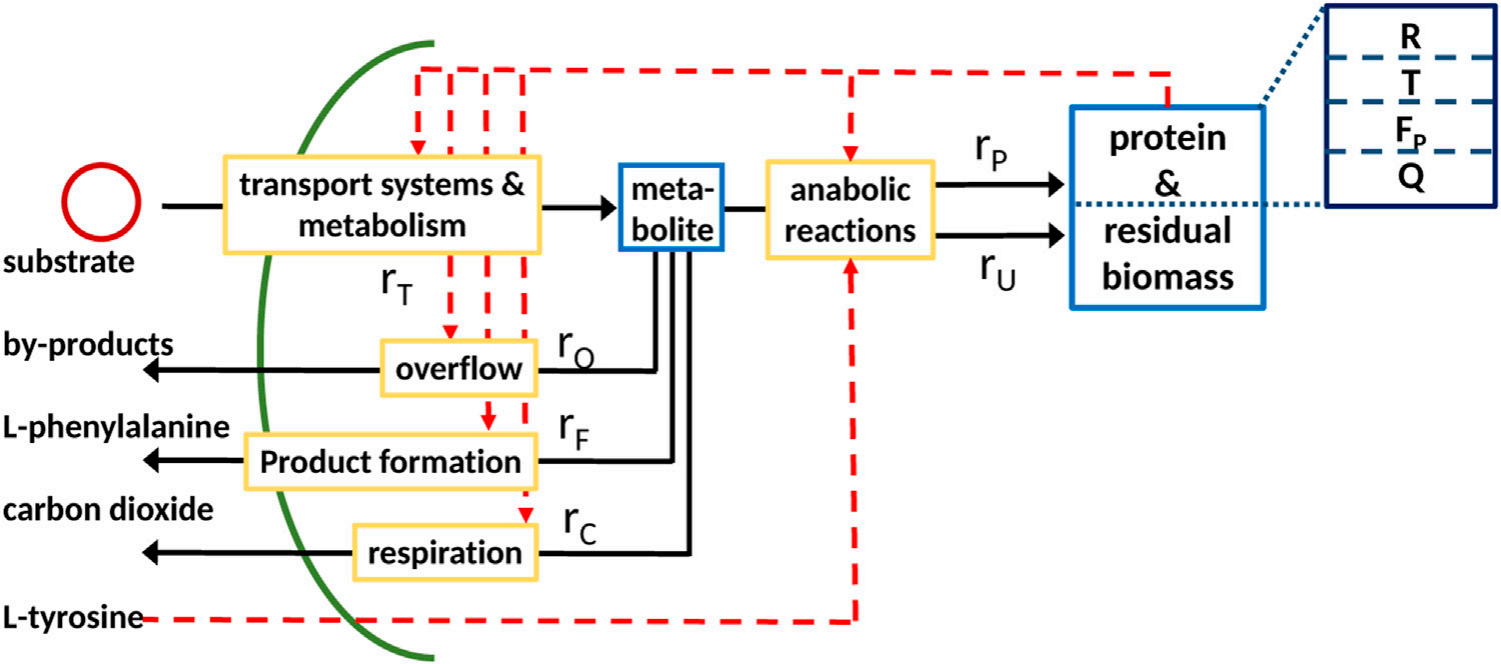
Scheme of the coarse-grained model expanded to include the formation of L-phenylalanine, respiration *r*_*C*_, and residual biomass *U*.

The proposed coarse-grained model approach can be used to model a biotechnological production process. In the scope of this research, we consider an L-phenylalanine producing *Escherichia coli* strain with glycerol as the substrate and decoupled biomass and product formation due to L-tyrosine auxotrophism, meaning biomass is only formed if L-tyrosine is available (Sprenger (2007), Weiner et al. (2014)). A more detailed description of the strain used can be found in the *Material and Methods* section. The L-phenylalanine production process is considered here as an example for a bioreactor production process. Due to the nature of coarse-grained models, the resulting model can easily be adapted to depict other production processes. The basic model is extended to include an additional rate *r*_*F*_ describing L-phenylalanine formation and a corresponding protein sector F as seen in Figure 5. Furthermore, we consider respiration implemented as rate *r*_*C*_ and the residual biomass fraction *U*.

The system (Eqs 2–4) is represented as follows:

$$N\;\;=\;\;\left(\begin{array}{cccccc} \alpha & -\gamma & -1 & -\delta & -2 & -1\\ 0 & \beta & 0 & 0 & 0 & 0\\ 0 & 0 & 0 & \epsilon & 0 & 0 \end{array}\right)$$ (24)

with the differential equations

$$\dot{\left(\begin{array}{c} M\\ P\\ U \end{array}\right)}\;\;=\;\;N\;\;\left(\begin{array}{c} r_{T}\\ r_{P}\\ r_{O}\\ r_{U}\\ r_{F}\\ r_{C} \end{array}\right)\;-\;\mu \;\;\left(\begin{array}{c} M\\ P\\ U \end{array}\right).$$ (25)

The next step is to determine the reaction rates. The rates concerning the central metabolism and overflow remain the same as in the basic model (Eq. 21). The L-phenylalanine production rate is dependent on the *F* fraction and the respiration rate *r*_*C*_ on the *T* fraction as it is part of the central metabolism. In order to incoporate the L-tyrosine auxotrophism, the protein synthesis rate *r*_*P*_ and synthesis rate of residual biomass *r*_*U*_ are modified to be multiplied with a function which is 1 if L-tyrosine is available and otherwise set to a low value of 0.1, corresponding to a low biomass formation during this phase, since it cannot be practically ensured that no L-tyrosine is available during this phase. This leads to the following set of reaction rates

$$\begin{array}{ll} r_{T}\;\; & =\;\;k_{T}\;\;\frac{S}{S+K_{T}}\;\;T\\ r_{P}\;\; & =\;\;k_{P}\;\;\frac{M}{M+K_{P}}\;\;R\;\;\tau \left(A\right)\\ r_{O}\;\; & =\;\;k_{O}\;\;M\;\;T\\ r_{U}\;\; & =\;\;k_{U}\;\;\frac{M}{M+K_{U}}\;\;R\;\;\tau \left(A\right)\\ r_{F}\;\; & =\;\;k_{F}\;\;M\;\;F_{P}\\ r_{C}\;\; & =\;\;k_{C}\;\;M\;\;T\text{,} \end{array}$$ (26)

where *A* is the available L-tyrosine and

$$\tau \left(A\right)=\left\{\begin{array}{cc} 1,\; & A>0\\ 0.1,\; & A\leq 0\text{.} \end{array}\right.$$ (27)

As the reaction rates are determined by the composition of the proteome, we can take advantage of the observations from the previous section. The allocation of T and R in the proteome is given by the estimated linear function of *M* as seen in Figure 4. A part of the proteome is allocated to fraction *F* after induction, which is accomplished through the shift of biomass production to product formation due to lack of L-tyrosine in the feed at time *t*_*ind*_ with delay as follows:

$$F_{P}\;\;=\;\;\phi \left(t\right)\;\;F_{\text{max}}\;\;P,$$ (28)

where

$$\phi \left(t\right)\;\;=\;\;\frac{t-t_{ind}}{\left(t-t_{ind}\right)+t_{\phi }}.$$ (29)

The remaining protein fraction *Q* is not of further interest.

**FIGURE 6:**
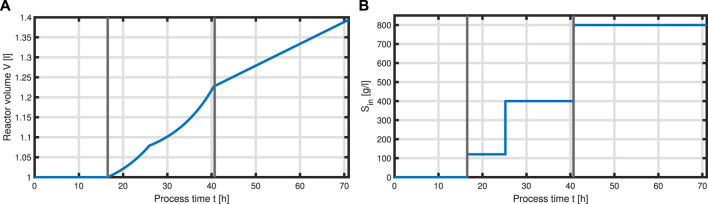
Feeding profile of the process. Bioeactor volume *V* **(A)** and substrate concentration (glycerol) of the feed *S*_*in*_ **(B)** over the time course of the process, where vertical lines indicate the three process phases (batch phase, fed-batch phase, and production phase with constant feeding).

**FIGURE 7:**
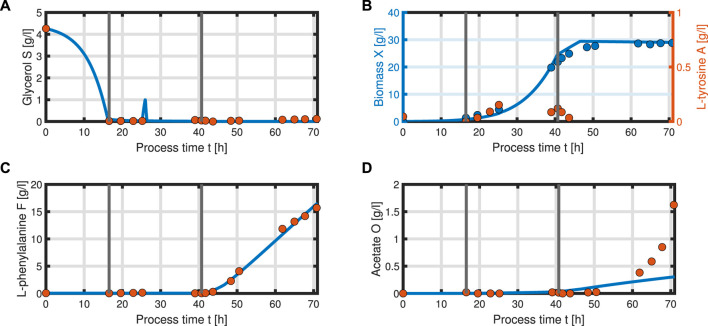
Comparison of the simulated quantities (solid blue line) against the experimental data (points) of the L-phenylalanine production process up to *t* = 71 h. Time course of the following concentrations: glycerol *S* **(A)**, biomass *X*, L-tyrosine *A* **(B)**, L-phenylalanine *F* **(C),** and acetate *O* as representative of the by-products of the process **(D)**. The data points of L-tyrosine, which are negative due to insufficient measurement sensitivity, are set to zero.

**FIGURE 8:**
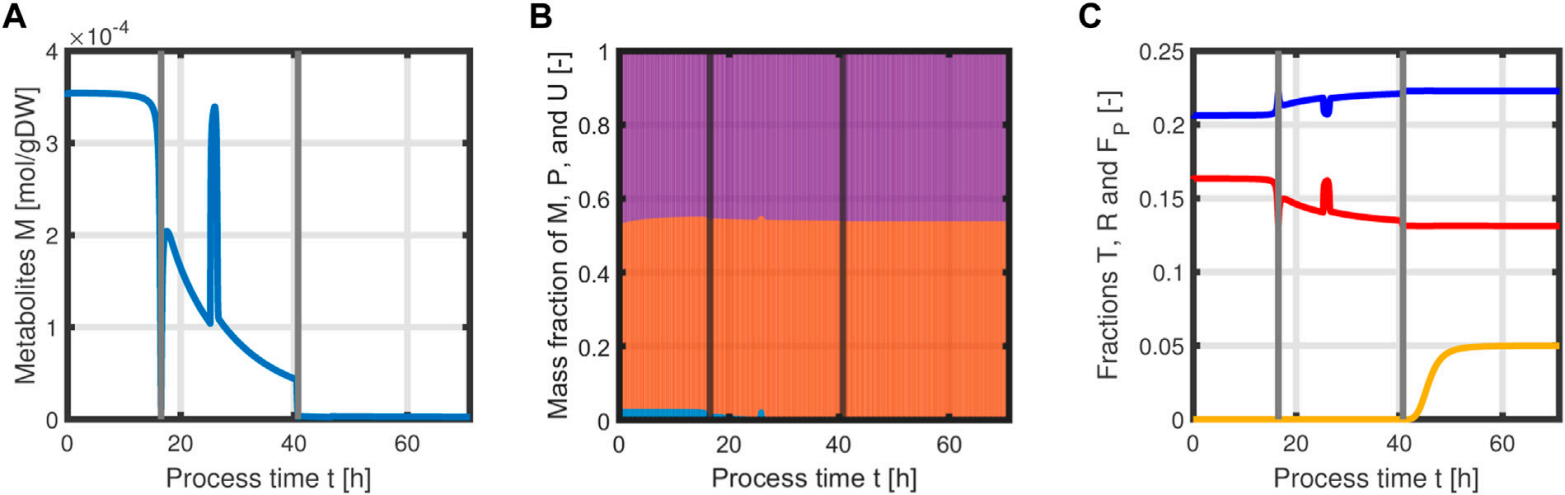
Time course of intracellular concentrations of the L-phenylalanine process: metabolites *M* **(A)**, mass fractions of proteins *P* (violet), and residual biomass *U* (orange) and metabolites *M* (blue), where *M* becomes negligible in the last process phase **(B)**. Mass fractions of *T* (blue), *R* (red), and *F* (yellow) over the course of the process **(C)**.

Now that we have formulated a system for the intracellular components forming the total biomass *X*, equations can be set up to model a complete bioprocess consisting of two process phases: a biomass production phase, followed by a batch phase, followed by two fed-batch phases with two different feeding solutions, and an L-phenylalanine production process phase which was initiated with induction of the cells with IPTG. For the process, we assume ideal mixing conditions in a bioreactor of the volume *V* with feeding rate *q*_*in*_ as seen in Figure 6​

$$\dot{V}\;\;=\;\;q_{in}$$ (30)

and only one feeding substrate (glycerol) *S* in [g/l] with feed substrate concentration *S*_*in*_

$$\dot{S}\;\;=\;\;\frac{q_{in}}{V}\;\;\left(S_{in}-S\right)\;-\;r_{T}\;\;X\;\;w_{S},$$ (31)

where the first term represents the ingoing substrate and dilution due to volume change and the second term the substrate uptake by the cells. The equation for the biomass *X* is given by

$$\dot{X}\;\;=\;\;\mu \;\;X\;-\;\frac{q_{in}}{V}\;\;X,$$ (32)

and the product equations are

$$\dot{F}\;\;=\;\;r_{F}\;\;X\;\;w_{F}-\;\;\frac{q_{in}}{V}\;\;F,$$ (33)

$$\dot{O}\;\;=\;\;r_{O}\;\;X\;\;w_{O}-\;\;\frac{q_{in}}{V}\;\;O,$$ (34)

with acetate *O* as the exemplary byproduct. Feeding profiles (Figure 6) determining the variables *q*_*in*_ and *S*_*in*_ and L-tyrosine concentrations during the process and initial values for the differential Eqs 30–34 are obtained from experimental data (see Supplementary Material). The bioprocess has been run in a stirred-tank bioreactor with a starting volume of *V*_0_ = 1 l; thus, we can that assume the environment in the bioreactor is well-mixed, and the description of the biomass by an average cell, as in the model presented, is sufficient. Experimental data suggest a stop in both biomass and product formation and a high accumulation of by-products after process time point *t* = 71 h (experimental data for the full process can be found in Supplementary Material). As reasons for this behavior have not been investigated at this point, the mechanistics to depict this are not incorporated in the model. Using Eqs 25–34, a numerical simulation up to process time *t* = 71 h was performed using MATLAB R2020a with ode15s as ordinary differential equation solver and was compared to experimental results (Figure 7). Analogous to the experiments, the simulation is divided into different process phases, and slightly different parameter sets were used for the biomass and L-phenylalanine production phase. The parameter set for the product formation phase contains higher reaction constants for byproduct formation and respiration. After each process phase, the solution of the end point was used as the initial value for the differential equations for the next process phase. Parameter values used for this simulation can be found in *Supplementary Materials*. Figure 7 shows good agreement between the simulated concentrations of substrate *S*, biomass *X*, L-phenylalanine *F*, and the experimental data. The peak of substrate concentration *S* during the fed-batch phase can be explained by the change of substrate concentration in the feed. The simulation of acetate concentration *O* shows the right trend, although not the exact behavior, of the by-products. Nevertheless, for the given parameter sets, the model can reproduce the overall dynamics of the L-phenylalanine production process. Besides the measurable quantities, the model can provide the intracellular concentrations as seen in Figure 8B, where the macromolecules *P* and *U* make up most of the biomass with both occupying nearly half of the biomass, and the mass fraction of the metabolites is negligible compared to that of the macromolecules, especially in the product formation phase. With a closer look at the allocation of the proteome, the fraction *R* follows the behavior of the metabolites *M*, decreases over the course of the process, and remains at a constant level during the product formation phase with fraction *T* forming the counterpart. The fraction for product formation *F* follows the description of Eqs 28, 29.

**FIGURE 9:**
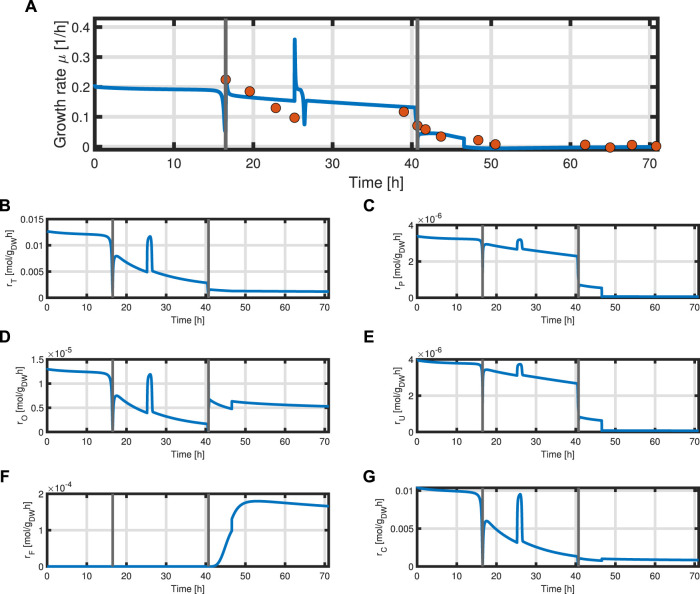
Time course of the simulated specific growth rate *μ* (blue) and the point-wise calculated specific growth rate obtained from experimental data indicated as orange dots **(A)**, substrate transport rate *r*_*T*_ **(B)**, protein synthesis rate *r*_*P*_ **(C)**, overflow metabolism rate *r*_*O*_ **(D)**, residual biomass synthesis rate *r*_*U*_ **(E)**, product formation rate *r*_*F*_ **(F),** and respiration rate *r*_*C*_ **(G)**.

In addition, the specific growth rate *μ* and the different reaction rates can be obtained from the model (Figure 9). The simulated growth rate *μ* roughly follows the trend of the growth rate pointwise calculated from the experimental data of biomass as seen in Figure 9A. The calculated growth rate has to be taken with caution as each point is calculated from two consecutive points with a large time difference and can heavily deviate from the actual growth rate. One can observe that due to the dependency of all rates on the metabolites *M* and the constant protein fraction *T*, all reaction rates follow the course of the metabolites during the biomass production phase (Figures 9B–D). In the product formation phase, the rates for the synthesis of all macromolecules deviate from the course of the metabolites as it is determined by the L-tyrosine auxotrophism (Figures 9C,D).

## 5 Optimal By-Product Secretion

The second example considers the optimal production of a metabolite (in this case *M*) that is excreted into the medium *via* reaction *r*_3_ (see Figure 1). Since *M* represents a metabolite from central metabolic pathways, it could stand for ethanol, acetate, or succinate which are all interesting biotechnological products. The stoichiometry and parameters are the same as in Eqs. 16–19. For the simulation study, and for a fair comparison of the outcoming results, the following conditions are fixed:

• A fed-batch process in a bioreactor is considered with a flexible input profile for the incoming substrate feed rate *q*_*in*_(*t*) as a function of time and a fixed-end time *t*_*end*_ = 20 h. With the feed, the substrate concentration can be adjusted in such a way that the metabolite is excreted at best. In contrast to a batch process, the substrate is fed into the medium and, therefore, high sugar concentrations in the beginning (as for the batch process) are avoided. Since a continuous process requires much more time, a steady state is reached normally first after five times the respective time constant (in our case approx. 20 h); this type of process design is also not considered here.
• The initial conditions are set fix for all model state variables.
• The bioreactor has a maximum working volume of 5 l, while in the beginning, the experiment starts with 1 l.
• The objective function is the amount of product expressed in mole at *t*_*end*_: *M*_*ex*_ *V*.

For the study, three different profiles are investigated. 1) A standard procedure, often applied in bioprocess engineering tries to feed the substrate in an exponential way to keep the specific growth rate *μ* constant. This requires that the substrate concentration in the bioreactor is nearly constant. The differential equation for the substrate *S* with function *q*_*in*_ and feed concentration *S*_*in*_ reads as follows:

$$\dot{S}\;\;=\;\;\frac{q_{in}}{V}\;\;\left(S_{in}\;-\;S\right)-r_{1}\;\;X,$$ (35)

and after setting this equation to zero, a function for *q*_*in*_ can be obtained:

$$q_{in}=\;\;\frac{r_{1}\;\;X\;\;V}{S^{in}\;-\;S};$$ (36)

with *m*_*X*_ = *X V* and a constant growth rate *μ*_0_ for this condition, we get

$$q_{in}=\;\;\frac{r_{1}\;\;m_{X0}\;\;e^{\mu_{0}\;\;t}}{S^{in}\;-\;S}.$$ (37)

Typically, *r*_1_ is estimated given the biomass yield coefficient *Y* during the batch phase and since the current substrate concentration is low (due to a small half-saturation value for substrate uptake), we finally obtain

$$q_{in}=\;\;\frac{\mu_{0}\;\;m_{X0}\;\;e^{\mu_{0}\;\;t}}{Y\;\;S^{in}}.$$ (38)

The feeding profile is applied after the end of the batch phase.

2) The second profile uses a polynomial function of time for the feeding rate:

$$q_{in}=\;\;\overset{4}{\underset{i=1}{\sum }}\;\;a_{i}\;\;t^{i},$$ (39)

with four parameters *a*_*i*_ to optimize.

3) The last profile is a piecewise linear profile with six fixed switching points *t*_*k*_:

$$q_{in}=\;\;q_{in}^{k}\;\;=\;\;const.\;\text{for}\;\;\;t_{k-1}\;\;\leq \;\;t\;\;\leq \;\;t_{k}.$$ (40)

Here, the values $q_{in}^{k}$ are the parameters that have to be optimized.

**FIGURE 10:**
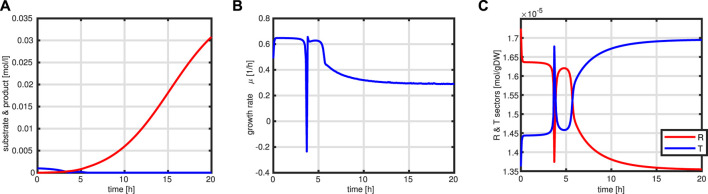
Simulation outcome for the standard fed-batch process. Time course of substrate (blue) and product (red) in the medium **(A)**. Growth rate *μ* **(B)**. Time course of the protein sectors *R* (red) and *T* (blue) **(C)**.

**FIGURE 11:**
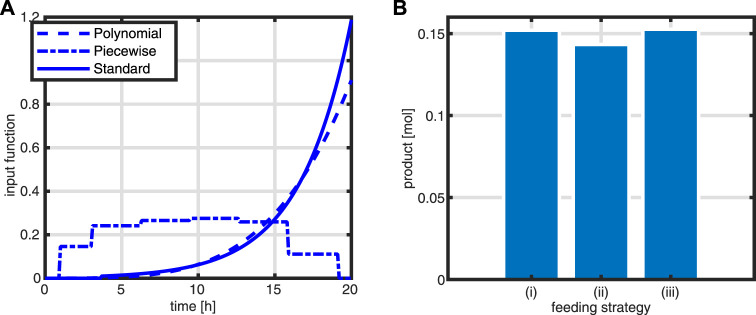
Comparison of the outcome of the three strategies. Time course for the feeding *q*_*in*_ as a function of time **(A)**; value of the objective function (from left to right: standard feeding, polynomial function, and piece-wise function) **(B)**.

First, a typical outcome for the standard case is shown in Figure 10. After 3.5 h, the substrate runs out and the feeding starts. The batch phase is characterized by very low productivity while the growth rate is at its maximum. In addition, during this phase, the *R* fraction is high (as already shown above) and, therefore, due to the coupling to the *T* fraction, the rate of byproduct formation is low. After starting the feeding, the intracellular system switches to high values for the *T* fraction Next, a comparison of the feeding profiles and the final value of the objective function is shown in Figure 11. Although the profiles are different, the final values of the objective function are comparable.

**FIGURE 12:**
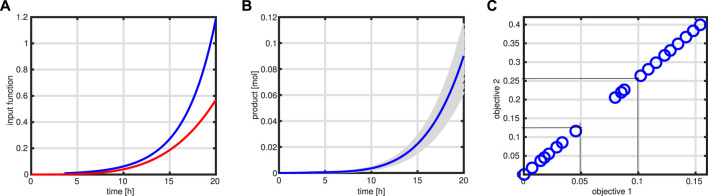
Robust optimization. Input profile (red) for the robust case in comparison to the standard case **(A)**. Product course of time for 100 simulations (in gray) with the variation in the maximal rate of the enzymatic processes and simulation with standard parameter (blue). **(B)**. Pareto front for two objective functions: product amount at time *t*_*end*_ and total amount of the substrate needed to produce the product. Thin lines indicate the cost change for an increase in product amount as described in the text **(C)**.

The simulation studies are based on a fixed set of kinetic parameters and, so far, did not consider any uncertainties with respect to the quality of these. Typically, kinetic parameters are obtained by parameter identification and subsequent parameter estimation and analysis. For our example at hand, in the next step, the uncertainty of the kinetic parameters is taken into account during the optimization procedure. Important parameters of the model are the maximal reaction velocities *r*_max_ for all reactions. To consider these uncertainties, an ensemble of 20 models is generated during each iteration step of the optimization. This results also in an ensemble for the values of the objective function Φ, following the approach proposed by Nimmegeers et al., (2016), and the objective function in this case is given by

$$\max\;\;\;E\left[\Phi \right]\;-\;\alpha \;\;\mathrm{Var}\left[\Phi \right],$$ (41)

with a weighting factor *α*, expectation E, and variance Var. With this formulation, stronger variations in the values of the objective function, expressed in the variance of Φ, are penalized. Since the feeding profile is geared to the growth rate, a much more conservative output is expected; if the substrate uptake, for example, would be higher than expected, more biomass would be produced, and few byproducts will be released. As can be seen in left of Figure 12, the input function (red curve) (and with this also the bioreactor volume) is lower than that in case of the standard procedure (blue curve). On the right side, different outcomes (in gray) for the variable product Pr are shown for 100 simulation runs, together with a simulation of the nominal values (blue curve).

Besides the optimization of the product at *t*_*end*_, also the cost of substrate is of interest. In a fed-batch process, the total amount of substrate *S*_*tot*_ fed, considering the substrate concentration at the beginning *S*_0_, can be calculated by

$$S_{tot}\;\;=\;\;\int_{t_{1}}^{t_{end}}\;\;q_{in}\left(t\right)\;\;S_{in}\;\;dt\;+\;S_{0}.$$ (42)

The outcome of having two objective functions, maximization of product at *t*_*end*_, and minimization of the substrate cost for the entire process can be presented with a Pareto front that is shown in Figure 12. As can be seen, a nearly perfect linear relationship is detected (it is to be noted that both objective functions have unit mole); increasing the product, for example, for 0.05 mol units results in cost for the substrate of 0.13 mol. In this way, an economic assessment of the process is possible.

## 6 Discussion

In bioprocess engineering, the design of experiments often is based on a mathematical description. While simple growth models taking into account only biomass, substrate, and product often are insufficient to describe the observed dynamics, whole-cell models are cumbersome and are difficult to calibrate. A good model comprises coarse-grained models because they are simple in the model structure but take into account the most important cellular processes. In this study, we propose an approach to use coarse-grained models based on a strict mass conservation to model bacterial growth as a basis for metabolic engineering applications. In this way, classical flux analysis could be extended to take into account fluxes into macromolecules such as the proteome. Additional solutions are provided by the null space of the mass matrix *W* that requires information on the mass fraction of the components of the model. With a simple example, we could show that depending on the mass composition of the cell, larger differences in the flux distribution in comparison to the standard approach could appear. With the condition of strict mass conservation, we are also able to provide a general solution for a cellular network independently of its internal structure.

As the focus of this research is coarse-grained models, we provided a formulation for a minimal model whose structure is in accordance with that in previous studies Sharma et al. (2018), Bertaux et al. (2020). Steady-state solutions are determined based on measured data (Schmidt et al., 2016) for the molecular composition of the cell. Especially, we consider two main protein fractions, an *R* fraction representing the transcription and translation apparatus and a *T* fraction, taking into account metabolic and transport enzymes. The outcome of these simulation studies is compared with that of a model where the *R* fraction is an adjustable quantity in an optimization program (the sum of the *R* fraction and *T* fraction is taken as constant). The results of the optimization program show that an optimal allocation of proteins led to a slightly higher growth rate with a comparable course of the *R* fraction as a function of the specific growth rate, and we conclude that the measured data are in good agreement with the expectation of an efficient and optimal acting organism.

Many studies have dealt with the derivation of growth laws under various conditions Klumpp et al. (2013), Bosdriesz et al. (2015), Hui et al. (2015). Based on the structure of the minimal model, we have expanded the model to include the dynamic environment in a bioreactor system that allows us to realize also different process design strategies such as feeding or continuous culture. Experimental data from an L-phenylalanine production process are taken as an example for parameter identification and estimation, and a good agreement between simulation and experimental data is obtained. A different design problem was addressed by finding optimal input profiles if the production of a metabolite from central pathways is of interest. Here, also, parametric uncertainties can be taken into account that leads to a much more conservative input profile. To summarize, coarse-grained models are a sound basis for the development of bioprocesses due to their simple structure with only a minor number of parameters and the flexibility to simulate and optimize different biotechnological process designs.

## Material and Methods for Experimental Cultivation of Triple Reporter Strain

### Strain

For the L-phenylalanine production process, in a stirred-tank bioreactor of 3.6 l working volume, a recombinant *Escherichia coli* FUS4 (pF81_kan_) strain was used as described by Gottlieb et al. (2014). This is a genetically modified strain with auxotrophies for L-phenylalanine and L-tyrosine by deletion of the chromosomal genes *aroF*, *pheA*, and *tyrA* (decoding for a DAHP synthase, bifunctional chorismate mutase / prephenate dehydratase, and a t-protein, respectively) along the aromatic biosynthesis pathway. Simultaneously, it harbors the pF81_kan_ plasmid decoding for the genes *aroF*, *pheA*, *aroB* (3-dehydroquinate synthase), and *aroL* (shikimate kinase 2) under the control of an inducable *tac* promoter. Furthermore, kanamycin resistance is integrated as the selection marker Gottlieb et al. (2014), Weiner et al. (2014).

### Cultivation Media

The cells were cultivated in a defined minimal medium with glycerol as the sole carbon source. All the components with their corresponding concentrations as well as its production protocol were adapted from the study by Weiner et al. (2014).

### Preculture Strategy

Provision of cell biomass for the inoculation for the cultivation in a stirred-tank bioreactor was realized by a two-step preliminary cultivation in shake flasks. First, a single colony of cells grown on minimal medium agar plates ( > 66 h at 37°C) was picked for inoculation of a single 100-ml shake flask with 10 ml minimal medium and cultivated at 37 °C and 150 rpm in an orbital shaker (Multitron, Infors HT, Switzerland) for 24 h. Afterward, the cells were transferred for further cultivation in two 500-ml shake flasks with 100 ml minimal media each and a starting optical density at 600 nm (OD_*600*_) of 0.01. After incubation at 37°C and 250 rpm for at least 24 h, the cells were centrifuged (4,500 rpm, 10 min) and resuspended in fresh minimal medium. These cell suspensions were used for inoculation of cultivations in the stirred-tank bioreactor with a starting OD_*600*_ of 0.1.

### Bioreactor Cultivation

For laboratory-scale cultivation of recombinant *E. coli* FUS4 (pF81_kan_) for L-phenylalanine production, a 3.6 glass stirred-tank bioreactor was used (Infors HT, Switzerland). The bioreactor was equipped with two six-bladed flat-blade turbines and three equidistant baffles. The minimal medium for cultivation was prepared *ex situ* and pumped into the bioreactor under sterile conditions to a starting volume of 1 l. The temperature was kept at 37 °C. 42*%* phosphoric acid and 25*%* ammonia were used as titration solutions to keep the pH at 7 ± 0.1. Dissolved oxygen levels above 30*%* were provided by step-wise increase of either stirrer speed or aeration rate up to 1,500 rpm and 5 l/min, respectively. Furthermore, an antifoam probe was used for controlled titration of antifoam solution, if necessary (AF204, 1:10 diluted, Sigma Aldrich, United States). The cultivation started with an initial batch phase. After depletion of glycerol, an exponential feeding was set for the biomass production phase with a defined growth rate of *μ*_set_ = 0.1 1/h. Two fed-batch media were used with either 120 g/l glycerol, 2.5 g/l L-phenylalanine, 3.6 g/l L-tyrosine, 60 g/l ammonium sulfate, and 0.1 g/l kanamycin (fed-batch medium 1) or 400 g/l glycerol, 1.11 g/l L-phenylalanine, 3.8 g/l L-tyrosine, 25 g/l ammonium sulfate, and 0.1 g/l kanamycin (fed-batch medium 2). The former and the latter were titrated with 25*%* ammonia or 5 M potassium hydroxide to allow complete dissolution of L-tyrosine. After a sufficiently high biomass concentration of at least 20 g/l was provided, the cells were induced with 0.3 mM IPTG. Fed-batch medium 3 with the components 800 g/l, 8 g/l ammonium sulfate, 8 g/l ammonium phosphate, and 0.1 g/l kanamycin was then constantly provided with a rate of 0.18 g_glycerol_/g_Biomass_h. At the start of each fed-batch media supply, the concentrated media components calcium chloride dihydroxide (15 g/l) and iron(II) sulfate heptahydrate with sodium citrate dihydrate (22.5 g/l and 200 g/l), magnesium sulfate heptahydrate (300 g/l), and thiamine hydrochloride (7.5 g/l) were mixed in a 1:5:1:1 ratio and injected to the fermentation broth *via* a septum. For the start of fed-batch phase 1, 2, or 3, a mixture of 4.8, 9.6, and 8.8 ml were injected, respectively Weiner et al. (2014).

### Analytics

Cell dry weights were measured gravimetrically. Pre-weighted dried 2-ml microcentrifuge tubes (80 °C for at least 24 h) were used for centrifugation of 2 ml of the cell suspension (21130 × g, 20 min, 4°C). The supernatant was further used for sample preparation for high-performance liquid chromatographies (HPLCs) or discarded. The cell pellet was dried again at 80°C for at least 24 h. The biomass concentration was calculated by the difference of weight between the microcentrifuge tube with dried cell pellets and the empty microcentrifuge tube. For the quantification of the amino acid concentrations of L-phenylalanine and L-tyrosine as well as for the organic compounds glycerol, acetate, lactate, succinate, pyruvate, malate, and ethanol, two different HPLCs were used. The samples for HPLC analysis were prepared by filtration of the supernatant of each sample through a 0.2-*μ*m filter and were stored at 4°C upon measurement. The quantification method for both amino acids is already described by Weiner et al. (2014) and was adapted from there. The organic compounds were quantified using a HPLC (Prominence-i LC-2030C, Shimadzu, Japan) with an ion-exchange column (Aminex HPX-87H 300 mm × 7.8 mm, Bio-Rad, CA, United States) and a refractive index detector (RID-20A, Shimadzu, Kyoto, Japan). 10 *μ*l of samples was injected to an isocratic flow of 0.6 ml/min and 5 mM sulfuric acid as mobile phase with a constant temperature of 60°C. The quantification of each component was realized by measurement of external standards.

## Data Availability

The original contributions presented in the study are included in the article/Supplementary Material; further inquiries can be directed to the corresponding author.

## Footnotes

1 Please note that underlined symbols are used for vectors.

2 Stoichiometric coefficients are given with Greek symbols.

## Supplementary Material

The Supplementary Material for this article can be found online at: https://www.frontiersin.org/articles/10.3389/fmolb.2022.806213/full#supplementary-material And the used MATLAB files in a repository under the following link: https://sourceforge.net/projects/coarse-grained-modeling/
