# Supplementary material for: Applications of Coarse-Grained Models in Metabolic Engineering
Source: Front Mol Biosci. 2022 Mar 8;9:806213. doi: 10.3389/fmolb.2022.806213 (PMC8957914; doi:10.3389/fmolb.2022.806213)
Supplement: Supplementary file 1 [file DataSheet1.pdf]

Supplementary Material

1 SUPPLEMENTARY INFORMATION

1.1 Mass Balance Equations and Translation Into ODEs

It is generally accepted to rearrange the left hand side of a mass balance $\dot{m}$ in the following way:

$$m \;=\; c\,V \;\Rightarrow\; \dot{m} \;=\; \dot{c}\,V \;+\; c\,\dot{V} \tag{S1}$$

with V is the reference state variable. In case of the reactor V is the reactor volume, and in case of the cellular system V is the cell volume that is replaced by the cell mass.

The volume V of the reactor system changes due to feeding q_{in} (we consider only one feeding) and outflow q from the reactor:

$$\dot{V} \;=\; q_{in} \;-\; q \tag{S2}$$

Substrate S is considered and its concentration c_S is based on the reactor volume V. Since reaction systems are considered, the number of molecules n is a more appropriate variable. Fortunately, the relation between mass m and number n is given by a fixed number, the molecular weight w, and one obtains:

$$\dot{n}_S \;=\; q_{in}\,S_{in} \;-\; q\,S \;-\; r_S^{**}\,, \tag{S3}$$

with the concentration is defined as $S \;=\; n_S/V$ and the uptake rate r_S^{**} is given in mol/h. Especially in biotechnology, quantity (dry) biomass m_X is of fundamental interest and a mass balance equation is used to describe the course over time by using the specific growth rate μ. By definition, μ describes the change of time of biomass divided by the biomass itself under batch conditions, and is the most important indicator for the quality of a process. From a formal point of view, biomass only can change, if nutrients are taken up or by-products are released; however, this poses the question or problem to know all substances in the medium that are taken up or excreted over time. Therefore, it is an accepted strategy to use the specific growth rate μ as cumulative parameter. Next, we will show in which way this is connected to the bio-chemical reaction network. The equation for the biomass reads:

$$\dot{m}_X \;=\; -\,q\,X \;+\; \mu\,m_X \tag{S4}$$

The last quantity of interest is a compound or a metabolite M that is inside the cell and therefore, its mass in the reference system will change if (i) biomass is leaving the reactor and (ii) by bio-chemical reactions that take part inside the cell. In this case, it is convenient to use the cellular dry weight as reference $M \;=\; n_M/m_X$, and therefore, the concentration for intracellular components has unit [mol/g DW]. Accordingly the mass balance read:

$$\dot{n}_M \;=\; -\,q\,M\;X \;+\; \sum_i r_i^{**} \tag{S5}$$

with the first term on the right side describes the mass flow of the biomass – with component M inside – out of the reactor and the sum term considers all reactions where M is involved. Now, we apply the procedure

from above to reformulate this equation in terms of the concentration. For the left side, we get as above:

$$\dot{n}_M \;=\; \dot{M}\, m_X \;+\; M\, \dot{m}_X \tag{S6}$$

that must be equal to the right hand side of the equation above. Rearranging the left hand side leads to an equation for $\dot{M}$ and with some basic calculations using the equation for the biomass from above, we result in:

$$\begin{aligned} \dot{M} &= \frac{1}{m_X}\left(-\, q\, M\, X \;+\; \underline{n_i}^T \underline{r}^{**} \;-\; M\, \dot{m}_X\right) \\ &= \frac{1}{m_X}\left(\underline{n_i}^T \underline{r}^{**} \;-\; \mu\, M\, m_X\right) \;=\; \underline{n_i}^T \underline{r} \;-\; \mu\, M \end{aligned} \tag{S7}$$

with $\underline{r}$ is the specific rate vector, that is, the rate vector $\underline{r}^{**}$ based on the biomass with unit [mol/gDW h]. If we write down the equations for all metabolites, then all single row vectors are combined in the stoichiometric matrix N

$$\underline{\dot{c}} \;=\; N\, \underline{r} \;-\; \mu\, \underline{c}, \tag{S8}$$

where $\underline{c}$ contains all metabolites. For the other quantities of interest, we proceed analogously and our final set of equations for biomass X and substrate concentration S reads:

$$\begin{aligned} \dot{S} &= D\;(S_{in} \;-\; S) - \underline{n}_S^T\; \underline{r} \\ \dot{X} &= (\mu - D)\;\, X \end{aligned} \tag{S9}$$

with the dilution rate $D = q/V$ and $\underline{n}_S^T$ the stoichiometric coefficients for substrate uptake.

Obviously, the equation system is not yet consistent, since the growth rate μ is still present. As already said, the change of mass of all cells should only depend on uptake and excretion of nutrient fluxes. To make the relation clear, we exploit a different way to write down the change of mass of all cells. Assume that in our vector of metabolites, the complete mass of the cell is represented, that is:

$$m_X \;=\; \sum_i m_{Mi} \;\Rightarrow\; \dot{m}_X \;=\; \sum_i \dot{m}_{Mi} \;=\; \sum_i \dot{n}_{Mi}\; w_i \tag{S10}$$

Plugging in all terms for $\dot{n}_{Mi}$ from above and comparing with Equation (S4) reveals an expression for the specific growth rate with the property of strict mass conversion:

$$\mu \;=\; \underline{w}\; N\; \underline{r} \tag{S11}$$

with vector $\underline{w}$ of all molecular weights.

1.2 Steady-state Analysis

In order to investigate the steady states of (S8), rewriting (S8) after plugging in (S11) leads to

$$\underline{\dot{c}} \;= (\mathrm{Id} \;-\; \underline{c}\; \underline{w}^T)\; N\; \underline{r} \;=\; W\; N\; r \tag{S12}$$

The steady states therefore has to be in the kernel of WN. The elements of the kernel of N is a subset of the kernel of WN and are defined by

$$\underline{r}_{n,0} = (\mathrm{Id} - N^{+}N)\ \underline{a}$$

where $\underline{a}$ is an arbitrary vector with the same dimension as the rate vector $\underline{r}_0$, using the Moore-Penrose inverse. The remaining elements of $\ker(WN)$, which we denote as $\underline{r}_{w,n,0}$, satisfy

$$WN\underline{r}_{w,n,0} = 0 \quad \text{and} \quad N\underline{r}_{w,n,0} = \underline{c}_{w,0} \neq 0$$

This means $\underline{c}_{w,0} \in \ker(W)$. To obtain $\underline{r}_{w,n,0}$, one can calculate the solution using the Moore-Penrose inverse again if we have the kernel of W

$$\underline{r}_{w,n,0} = N^{+}\underline{c}_{w,0} + (\mathrm{Id} - N^{+}N)\ \underline{a}$$

We observe that the last term is the same as the kernel of N. Thus, the previous equation determines the general solution of the kernel of WN

$$\underline{r}_{w,n,0} = \underline{r}_0.$$

The next step, is to calculate the kernel of W

$$W \;=\; \mathrm{Id} \;-\; \underline{c}\ \underline{w}^T \;=\; \mathrm{Id} \;-\; \begin{pmatrix} c_1w_1 & \cdots & c_1w_n \\ \vdots & \ddots & \vdots \\ c_nw_1 & \cdots & c_nw_n \end{pmatrix}. \tag{S13}$$

Let n be the dimension of W, corresponding to the number of components in the network. The determinant of W can be easily calculated up to dimension 3 and for larger dimensions obtained from the Leibniz formula, leading to

$$\det(W) \;=\; 1 \;-\; \sum_i w_i c_i \tag{S14}$$

which is zero due to strict mass conservation as the latter term equals 1. One can show furthermore, that the kernel of W is one-dimensional, as the values of W have a positive sign on the diagonal and negative sign otherwise

$$\begin{pmatrix} + & - & \cdots & - \\ - & + & \cdots & - \\ \vdots & & \ddots & \vdots \\ - & \cdots & - & + \end{pmatrix}. \tag{S15}$$

The rank of the matrix W is given by the maximum number of linearly independent column or row vectors of the matrix. As the n vectors of W all have exactly one positive entry at a different position of the vector, one needs n vectors in order to find a linear combination to form zero and the maximum number of linearly independent vectors is $n-1$. This argumentation is true for both the set of column and row vectors. In

order to obtain the linear combination, each equation of the system can be written as

$$0 \;=\; \lambda_i \;-\; c_i \sum_k \lambda_k w_k \tag{S16}$$

If we choose $\lambda_i = sc_i$ with $s \in \mathbb{R}$, then the set of equations hold as the latter term forms

$$\sum_k s\ c_k w_k \;=\; s \sum_k c_k w_k \;=\; s. \tag{S17}$$

Thus, the kernel of W is

$$r_{w,0} \;=\; s\ \underline{c}, \qquad s \in \mathbb{R}. \tag{S18}$$

1.3 Steady-state Analysis of the Minimal Model

In the example of the minimal model, N^+ is given by

$$N^+ = \begin{pmatrix} \frac{\alpha}{\alpha^2+1} & \frac{\gamma\alpha}{\beta+\beta\alpha^2} \\ 0 & \frac{1}{\gamma} \\ -\frac{1}{\alpha^2+1} & -\frac{\gamma}{\beta+\beta\alpha^2} \end{pmatrix}. \tag{S19}$$

Therefore, we obtain

$$Id - N^+N = \begin{pmatrix} \frac{1}{\alpha^2+1} & 0 & \frac{\alpha}{\alpha^2+1} \\ 0 & 0 & 0 \\ \frac{\alpha}{\alpha^2+1} & 0 & \frac{\alpha^2}{\alpha^2+1} \end{pmatrix}. \tag{S20}$$

With this formulation, we can see that $\mathrm{rank}(\mathrm{Id} - N^+N) = 1$, leading to a one-dimensional solution space for r. The image of $\mathrm{Id} - N^+N$ is given by

$$\mathrm{span}\left(\begin{pmatrix} \frac{1}{\alpha^2+1} \\ 0 \\ \frac{\alpha}{\alpha^2+1} \end{pmatrix}\right). \tag{S21}$$

2 SUPPLEMENTARY TABLES AND FIGURES

2.1 Figures

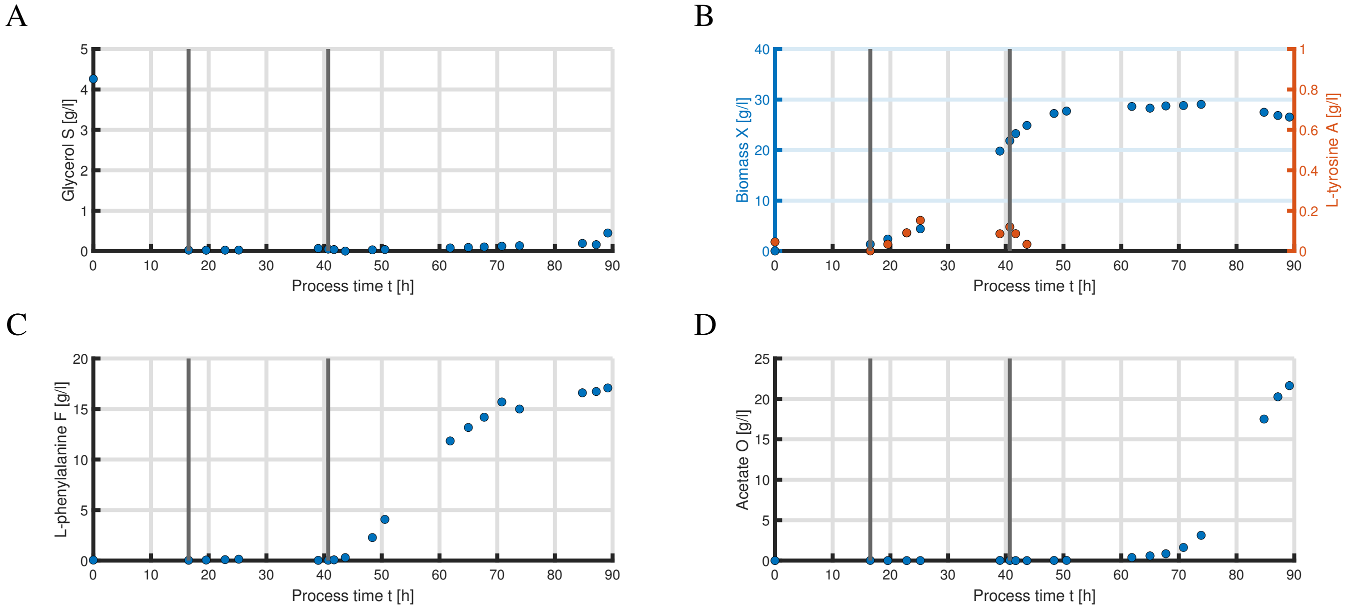

Figure S1: Full L-phenylalanine production process: time course of glycerol S (A), biomass X, L-tyrosine A, where negative values due to insufficient measurement sensitivity are set to zero, (B), L-phenylalanine F (C) and acetate O as exemplary by-product of the process (D).

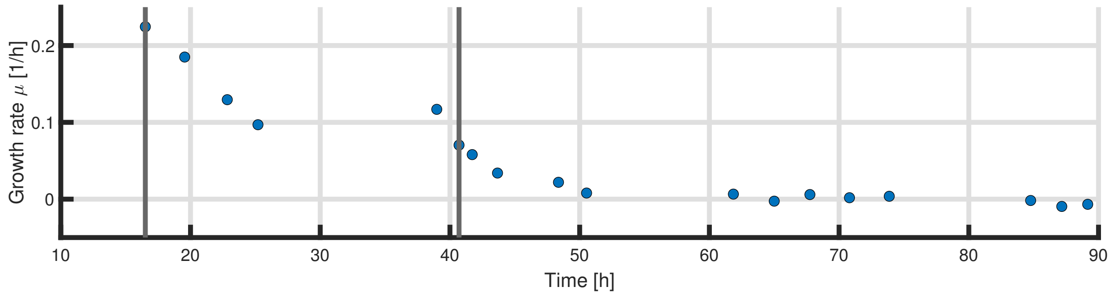

Figure S2: Full L-phenylalanine production process: time course of pointwise calculated specific growth rate μ.

2.2 Tables

Table S1. Overview of parameters values used for the simulation of a production process

Parameter	Description of parameter	Value	Unit
V_0	Initial reactor volume	1	l
X_0	Initial biomass concentration	0.0333	g_{DW}/l
S_0	Initial substrate concentration	4.2609	g/l
M_0	Initial metabolite concentration	$3.5 \cdot 10^{-4}$	mol/g_{DW}
P_0	Initial protein concentration	$1.7760 \cdot 10^{-5}$	mol/g_{DW}
U_0	Initial residual biomass concentration	$1.8926 \cdot 10^{-5}$	g_{DW}/l
F_0	Initial L-phenylalanine concentration	0.0495	g/l
O_0	Initial acetate concentration	0	g/l
F_{max}	Maximal fraction of L-phenylalanine proteins	0.05	-
ψ_P	Protein length	300	-
w_S	Molecular weight of substrate	92.09	g/mol
w_M	Molecular weight of metabolites	88.06	g_{DW}/mol
w_P	Molecular weight of proteins	$w_M \cdot \psi_P$	g_{DW}/mol
w_U	Molecular weight of residual biomass	$w_M \cdot \psi_P$	g_{DW}/mol
w_F	Molecular weight of L-phenylalanine	165.19	g/mol
k_T	Kinetic constant for transport rate	$3.5 \cdot 10^3$	1/h
k_P	Kinetic constant for protein synthesis rate	1.2	1/h
$k_{O,b}$	Kinetic constant for overflow metabolism rate (biomass production phase)	$1 \cdot 10^4$	g/molh
$k_{O,p}$	Kinetic constant for overflow metabolism rate (production phase)	$5 \cdot 10^5$	g/molh
k_U	Kinetic constant for residual biomass synthesis rate	1.4	1/h
k_F	Kinetic constant for L-phenylalanine production rate	$7 \cdot 10^7$	g/molh
$k_{C,b}$	Kinetic constant for respiration rate (biomass production phase)	$8 \cdot 10^6$	g/molh
$k_{C,p}$	Kinetic constant for respiration rate (production phase)	$8 \cdot 10^7$	g/molh
K_T	Michealis-Menten constant for transport rate	0.05	g/l
K_P	Michealis-Menten constant for protein synthesis rate	$0.1 \cdot 10^{-4}$	mol/g_{DW}
K_U	Michealis-Menten constant for residual biomass synthesis rate	$0.1 \cdot 10^{-4}$	mol/g_{DW}
α	Stoichiometric coefficient for transport rate	1	-
β	Stoichiometric coefficient for protein synthesis rate	1	-
γ	Stoichiometric coefficient for protein synthesis rate	300	-
ϵ	Stoichiometric coefficient for residual biomass synthesis rate	1	-
δ	Stoichiometric coefficient for residual biomass synthesis rate	300	-
